# The difficulty in defining right ventricular failure at the bedside and its clinical significance

**DOI:** 10.1186/s13613-021-00912-7

**Published:** 2021-08-05

**Authors:** A. Vieillard-Baron, M. R. Pinsky

**Affiliations:** 1 Medical Intensive Care Unit, Ambroise Paré Hospital, AP-HP, Boulogne-Billancourt, INSERM UMR 1018, Clinical Epidemiology Team, CESP, Paris-Saclay University, Villejuif, France; 2 Department of Critical Care Medicine, University of Pittsburgh, Pittsburgh, PA USA

Zhang et al. reported right ventricular (RV) function in 215 mechanically ventilated patients with septic shock [1]. They give us the opportunity to discuss and re-emphasize some key aspects of the bedside evaluation of RV function, as well as address unresolved questions.

The authors dissociated RV systolic dysfunction, defined by echocardiography as a tricuspid annular plane systolic excursion (TAPSE) below 16 mm or an RV fractional area changes below 35%, from RV failure defined by RV dilatation and elevated central venous pressure (CVP) [1]. While the criteria of defining RV systolic dysfunction are well-known and already described in septic patients [2], the concept of RV failure is more recent and still requires discussion. Besides, Vieillard-Baron et al. suggested that RV systolic dysfunction could be dissociated from RV failure, because they found no difference in TAPSE values whatever the combination of RV size and CVP [3]. Although RV systolic dysfunction can be created rapidly by elevations in pulmonary arterial pressure due to pulmonary embolism, hyperinflation, and pulmonary hypertension, RV failure has a more ominous clinical implication, since it may persist following reversal of increased pulmonary outflow pressures.

In different statements, experts proposed to define RV failure as a status associating “significant” RV dilatation to maintain an adequate cardiac output, when possible, with systemic congestion which may be assessed by CVP [4]. They more consider RV systolic function as an early remodeling state in the process of RV injury or in response to progressive pulmonary hypertension. While this approach is well-supported by physiology, recent papers including the one by Zhang et al. did not support it. If accurately defined, RV failure is expected to worsen outcome and Zhang et al. found that RV failure with normal echo parameters of systolic function was not associated with 30-day mortality, whereas the association of RV failure plus RV systolic dysfunction was [1]. Lanspa et al. previously published similar results, where RV systolic dysfunction was associated with 28-day mortality, while left ventricular (LV) systolic dysfunction was not [2].

Reasons for this apparent absence of validation of physiology are not obvious. RV dilatation is defined using echocardiography when the RV/LV end-diastolic area is > 0.6 and systemic congestion when CVP is ≥ 8 mmHg. Applying such a definition, Vieillard-Baron et al. found a 42% incidence of RV failure in mechanically ventilated septic patients [3] and Zhang et al. an almost similar incidence of 32% [1]. One could expect that when applying different thresholds for RV dilatation and CVP, an association with the outcome could be found. It was not the case in the paper by Zhang et al. but Vieillard-Baron et al. reported much higher median values for RV size and CVP in the group with suspected RV failure, 0.7 [0.7;0.9] and 12 mmHg [10;14], respectively, while they unfortunately did not evaluate the impact of RV failure on the outcome. The use of a CVP threshold of 8 mmHg for RV failure is probably too low in ventilated patients with PEEP for most of them because of the part related to the transmitted pressure. Mekonsto-Dessap et al. reported in 752 moderate-to-severe ARDS that only severe acute cor pulmonale, which means an RV/LV end-diastolic area > 1 with a paradoxical septal motion, was associated with in-ICU mortality, but CVP was not reported [5]. Furthermore, Simon et al. found that RV remodeling during pulmonary hypertension occurs with CVP values < 8 mmHg and only with RV dilation does CVP exceed 8 mmHg, with most RV dilation in patients having CVP > 12 mmHg [6]. An interesting point is that patients with RV failure are very frequently unresponsive to fluids whatever respiratory variations in pulse pressure, which could then represent a warning signal for intensivists that the right ventricle is in a bad shape [3].

Operationally, patients found to have RV dilatation and elevated CVP need to be assessed for evidence of pulmonary hypertension and volume overload. To the extent that systemic arterial pressure can be sustained greater than pulmonary artery pressures by either using systemic vasopressors or selective pulmonary vasodilators (e.g., inhaled NO, intravenous PGE or PGI) without the presence of systemic hypoperfusion (i.e., normal capillary refill time, urine output, and serum lactate), diuresis should be cautiously started to reverse RV dilation and improve RV systolic and diastolic function.

Finally, because of the absence of gold standard to define RV failure and of the very specific physiology of the right ventricle which includes initial dilatation with increases in CVP, defining RV failure at bedside with the best sensitivity and specificity is still a challenge. Current published papers are not clear and precise enough to give intensivists the most accurate definition. It should be elucidated in future studies, as it is now well-known that RV function is crucial to evaluate at the bedside to optimize respiratory and circulatory management of critically-ill patients.

## Data Availability

Not applicable.
